# Analysis of the state of sustainable development and key targets: A case study of China and “Belt and Road” countries

**DOI:** 10.1016/j.heliyon.2025.e42305

**Published:** 2025-01-28

**Authors:** Shun Chen, Ping Jiang, Pin Zuo, Zolboo Dashnyam

**Affiliations:** a Department of Environmental Science and Engineering, Fudan University, Shanghai, 200433, China; b Fudan Tyndall Centre, Fudan University, Shanghai, 200433, China; c School of International Relations and Public Affairs, Shanghai International Studies University, Shanghai, 200083, China; d Mongolia's Institute of International Affairs Research Center, Mongolian Academy of Sciences, Ulaanbaatar, 13330, Mongolia

**Keywords:** Sustainable development goals (SDGs), Belt and road initiative (BRI), Green development, Environmental governance, Sustainable development cooperation

## Abstract

This study examines the sustainable development progress of China, Kazakhstan, Mongolia, and Pakistan under the Belt and Road Initiative (BRI) from 2000 to 2019, using 63 indicators across 17 Sustainable Development Goals (SDGs). These countries were selected due to their significant energy reserves and their pivotal role in the BRI's green sustainable development goals. As major energy producers in China's vicinity, their cooperation in environmental governance and carbon neutrality offers a tremendous opportunity to align their sustainability efforts, directly contributing to both regional and global SDG targets. By applying data from the Sustainable Development Solutions Network (SDSN) and Bertelsmann Stiftung, and utilizing social network analysis and principal component analysis, the study identifies key SDGs and explores their interlinkages across these countries. The findings underscore the critical need for these nations to focus on environmental and social sustainability, fostering enhanced cooperation in green development. This research provides valuable insights for policymakers to formulate effective, data-driven green policies, promoting long-term sustainable development and cooperation within the BRI framework.

## Highlights

• Analyzes sustainable development trends and SDG implementation in four countries.
• Assesses the interconnectivity of SDGs in China, Kazakhstan, Mongolia, and Pakistan.
• Explores regional collaboration opportunities within the Belt and Road framework.
• Proposes a future research direction focused on post-2019 data and COVID-19 impacts.

## 1 Background introduction

The Belt and Road Initiative (BRI), proposed in 2013, focuses on the development of economic partnerships between China and countries along the route, leveraging bilateral and multilateral mechanisms and regional cooperation platforms. This initiative aims to foster a community of shared interests, destiny, and responsibilities [1,2]. A key aspect of the BRI is the shared commitment to sustainable development goals (SDGs), which create significant opportunities for collaboration in environmental governance, aligning well with the sustainable development objectives of each country.

Since the 1992 Rio Declaration on Environment and Development, the global sustainability agenda has gained significant momentum, driven by the need to address the socioeconomic and environmental impacts of rapid economic growth. In response to these challenges, 193 United Nations Member States adopted the 2030 Agenda for Sustainable Development and the SDGs in 2015. This landmark agreement marked a critical step towards addressing global issues such as poverty, inequality, and climate change [3]. The SDGs provide a universal framework for assessing progress across a broad range of environmental, social, and economic dimensions. By establishing clear targets, the SDGs enable data-driven evaluations that help policymakers identify areas for improvement and prioritize interventions. Moreover, the SDGs enhance international comparability and accountability, fostering collaboration to tackle pressing global challenges [4].

Over recent years, scholars around the world have examined sustainable development through the lens of SDGs. For example, Shibata and Wilson explored the relationship between poverty, education, and the development of health and sanitation facilities in the slums of East Indonesia, emphasizing that eradicating extreme poverty is crucial for achieving sustainable development [5]. Similarly, Roy and Pramanick analyzed the interconnection between water and sanitation facilities, health, and poverty eradication in India, showing that GDP growth positively impacts sanitation-related indicators and reduces waterborne diseases [6]. In Southern Africa, Charles et al. developed a baseline index for SDGs related to agriculture [7], while Craig and Robert highlighted potential conflicts between SDGs on poverty, inequality, food security, economic growth, and biodiversity [8]. These studies reflect the complexity of SDG implementation and the challenges of balancing competing goals. Likewise, numerous studies have focused on analyzing the implementation of specific measures to attain sustainable development goals, such as Akinlolu G. Omisore's research on achieving these goals in sub-Saharan Africa [9]. R Srikanth developed a policy framework that amalgamates all low-carbon energy technologies with coal, aiming to fulfill India's sustainable development objectives [10]. Ranjula and Amin facilitated the European Union's sustainable development via renewable energy sources [11].

Turning to the Belt and Road Initiative, most studies have primarily focused on its impact across different regions, with an emphasis on investment and cooperation in new energy technologies. Researchers such as Sarah Chan, Mario Esteban, and Mingjiang Li have analyzed the effects of the BRI in East Asia, Europe, and the Indo-Pacific region, respectively [[12], [13], [14]]. These studies underscore the potential for green cooperation within the BRI framework, particularly in the energy sector. However, they often overlook the broader interconnections between SDGs and the opportunities for environmental governance that the BRI can facilitate.

While research on SDGs has predominantly focused on individual countries or regions, studying the SDGs within the context of the BRI offers unique insights. As the BRI emphasizes green and sustainable development, understanding the interconnections between SDGs in participating countries becomes increasingly important. This study therefore aims to expand this research field by focusing on the SDG interconnectivity among Belt and Road countries, with a particular emphasis on China and its neighbors—Kazakhstan, Mongolia, and Pakistan. By integrating the SDGs into the BRI framework, this research seeks to identify key cooperation opportunities and challenges, which are essential for fostering long-term, sustainable development.

The paper evaluates the development status of these four countries in relation to 17 SDGs, using 63 indicators from 2000 to 2019. It utilizes data from the Sustainable Development Solutions Network (SDSN) and Bertelsmann Stiftung to analyze the sustainable development situation in these countries [15]. Social network analysis and principal component analysis (PCA) are employed to identify key SDGs and assess their interconnections. This approach allows for a deeper understanding of the progress each country has made toward achieving the SDGs and highlights potential areas for further collaboration, particularly in areas where SDGs align or conflict.

This study builds on the work of Feng et al. who also examined sustainable development in the Belt and Road countries. However, we address key gaps in their research, particularly in terms of time frame and geographic focus [16]. While Feng et al. focused on a shorter time period and did not emphasize SDG interconnectivity, our study spans from 2000 to 2019, providing a more comprehensive and up-to-date view of sustainable development trends [16]. Additionally, by selecting Kazakhstan, Mongolia, and Pakistan—energy-rich countries that share borders with China—we bring a fresh perspective to sustainable development within the Belt and Road context. These countries are not only central to the energy landscape of the BRI but also provide key insights into regional cooperation, particularly in environmental governance and green energy. Moreover, this study contributes to advancing the research field by examining how the SDGs in these countries are interconnected. By focusing on SDG interconnectivity, we move beyond isolated sectoral analyses and offer a more integrated view of how these nations can collaborate to achieve sustainable development goals, particularly in the context of energy and green development. This is an area that was not fully explored in Feng et al. which primarily focused on individual SDG indicators without addressing the synergies between them [16].

This study contributes to the field in several key areas: (1) it provides a detailed quantitative analysis of sustainable development scores, breaking down high-level SDG indicators into specific, measurable components; (2) it integrates the national contexts of China, Kazakhstan, Mongolia, and Pakistan, illustrating how these countries can collaborate within the Belt and Road framework to address sustainable development challenges; and (3) it proposes a scalable framework for future research, particularly in post-2019 analyses and expanding SDG cooperation among Belt and Road countries.

## 2 Research methodology

### 2.1 Social network analysis

Social Network Analysis (SNA) is a quantitative analysis method developed by sociologists based on mathematical methods and graph theory [17]. The term “social network” refers to the collection of social actors and the relationships among them [18]. It composed of social actors (nodes) and the myriad social relationships (edges) between them. SNA is a quantitative research method used to quantitatively analyze the relationships between individuals within social networks [19]. In social network analysis, “centrality analysis” is an analytical method for assessing the importance of members within a social network. By calculating individual centrality metrics, one can determine the level of importance of an individual within a social network [18].

In recent years, with the swift proliferation of the internet and big data, Social Network Analysis has found extensive applications in areas like social networks, energy resources, economics, the environment, and health and medical sectors [[20], [21], [22]]. Through social network analysis of 63 indicators of sustainable development goals, this article selects and confirms key objectives, thereby enhancing the realization of sustainable development targets. In this paper, each goal is considered as a node, and the causal relationships between nodes are defined as directed edges [23]. It also takes into account indicators such as each node's weighted in-degree, weighted out-degree, weighted degree, closeness centrality, betweenness centrality, and eigenvector centrality to assess the importance of nodes [24,25]^.^ The subset of goal data with the greatest impact on the overall network is identified through calculations using Gephi software [26]. In this study, the 63 sustainable development goals influence and constrain each other, creating complex relationships. By employing social network analysis methods, internal relations between sustainable development goals of different countries can be determined, thereby identifying key goals that have the greatest impact on sustainable development mechanisms.

### 2.2 Principal component analysis

Principal Component Analysis (PCA) was initially introduced by K. Pearson for analyzing non-random variables, and later H. Hotelling extended this method to the case of random vectors [27]. This method reduces high-dimensional data to lower dimensions by identifying major patterns of variation in the data, facilitating better understanding and interpretation. In SPSS software [28], PCA is performed on the 63 Sustainable Development Goal (SDG) indicators, yielding the principal components and the matrix of principal component score coefficients for each country's SDGs. Additionally, the first few indicators from principal components with a significant variance contribution rate (greater than 5 %) are selected as potential supplementary indicators. Moreover, supplementary indicators obtained from principal component analysis are incorporated into the target data subset, which is then analyzed using linear regression with the extracted principal components. The target subset's effect on the principal components can be interpreted through the R^2^ value, which measures the degree of fit. The extent to which the target subset explains the sustainable development goals dataset can be assessed by the product of R^2^ and the variance contribution rate of the principal components. If the level of explanation is below 85 % (a subjective estimate of depreciation), it necessitates the selection of additional objectives from the target subset. As more objectives are added, the regression calculations are repeated until the smallest set of objectives with an explanatory power exceeding 85 % is obtained [29]. Finally, variables are selected, determined, and optimized from these objective variables to identify the primary set of sustainable development goal indicators.

Calculation formulas:

(1) Standardization of Raw Data

Assume there are m indicator variables for principal component analysis: $x_{1},x_{2},\ldots ,x_{m}$, with n evaluation subjects. The value of the jth indicator for the ith evaluation subject is $x_{ij}$. Convert each indicator value $x_{ij}$ into the standardized indicator ${\text{ZX}}_{ij}$. The standardized values are calculated using Equation (1):

(1) $$ZX_{ij}=\frac{x_{ij}-\bar{x_{j}}}{s_{j}},\left(i=1,2,\ldots ,n;j=1,2,\ldots ,m\right)$$

where ${\text{ZX}}_{ij}$ is the value after standardization, and $\bar{x_{j}}$ and $s_{j}$ are the sample mean and standard deviation of the *j*th indicator, respectively.

(2) Establishment of Correlation Coefficient Matrix R

The correlation coefficient matrix $R={\left(r_{ij}\right)}_{m*m}$, as shown in Equation (2):

(2) $$r_{ij}=\frac{\sum_{k=1}^{n}ZX_{ki}*ZX_{kj}}{n-1},\left(i,j=1,2,\ldots ,m\right)$$

where $r_{ii}=1,r_{ij}=r_{ji,}r_{ij}$ is the correlation coefficient between the *i*th and *j*th indicators.

(3) Calculation of Eigenvalues and Eigenvectors of the Correlation Coefficient Matrix

Calculate the eigenvalues $\lambda_{1}\geq \lambda_{2}\geq \ldots \geq \lambda_{m}\geq 0$ and corresponding eigenvectors $a_{1},a_{2},\ldots ,a_{m}$ of the correlation coefficient matrix R, where $a_{j}={\left(a_{1j,}a_{2j},\ldots ,a_{nj}\right)}^{T}$. The eigenvectors form m new indicator variables.

(3) $$\begin{array}{l} P_{1}=a_{11}ZX_{1}+a_{12}ZX_{2}+\ldots +a_{1n}ZX_{n}\\ P_{2}=a_{21}ZX_{1}+a_{22}ZX_{2}+\ldots +a_{2n}ZX_{n}\\ \ldots \ldots \\ P_{m}=a_{m1}ZX_{1}+a_{m2}ZX_{2}+\ldots +a_{mn}ZX_{n} \end{array}$$

In formula (3), $P_{1}$, $P_{2}$, $P_{m}$ represents the principal component score, the new variable, expressed as matrix P = A ∗ X;

$$P=\left[\begin{array}{l} P_{1}\\ P_{2}\\ \ldots \\ P_{m} \end{array}\right],A=\left[\begin{array}{cccc} a_{11} & a_{12} & \ldots & a_{1n}\\ a_{21} & a_{22} & \ldots & a_{2n}\\ \ldots & \ldots & \ldots & \ldots \\ a_{m1} & a_{m2} & \ldots & a_{mn} \end{array}\right],X=\left[\begin{array}{l} ZX_{1}\\ ZX_{2}\\ \ldots \\ ZX_{n} \end{array}\right]$$

$P_{1}$, $P_{2}$, …, $P_{m}$ are the 1st, 2nd, …, *m*th principal component scores of $ZX_{1}$, $ZX_{2}$, …, $ZX_{n}$, respectively.

(4) Calculation of Cumulative Contribution Rate

The cumulative contribution rate is a key metric used to evaluate the proportion of total variance explained by the selected principal components. It is calculated using Equation (4):

(4) $$\partial_{s}=\frac{\sum_{k=1}^{s}\lambda_{k}}{\sum_{k=1}^{m}\lambda_{k}}$$

$\partial_{s}$ is the cumulative contribution rate of the principal component $P_{1}$, $P_{2}$, …, $P_{s}$. When $\partial_{s}$ approaches 1($\partial_{s}$ = 0.85,0.90,0.95), select the first s indicator variables $P_{1}$, $P_{2}$, …, $P_{s}$ as s principal components, replacing the original m indicator variables. Thus, a comprehensive analysis of the s principal components can be conducted.

In this study, PCA is employed to analyze the development trends of various countries in terms of sustainable development goals. By calculating the scores of each country on the principal components, the implementation of the main sustainable development goals can be quantitatively assessed. PCA is also used to test, prioritize, and supplement the key objective set identified by Social Network Analysis, thus more scientifically and reasonably selecting the critical sustainable development goals for each country. By identifying the relevancy and key sustainable objectives of the “Belt and Road” countries, a framework for promoting green sustainable development cooperation can be further constructed, providing more opportunities and possibilities for cooperation among countries.

### 2.3 Method of the Bertelsmann Stiftung's calculations

To ensure comparability across indicators, each variable was rescaled to a range from 0 to 100, where 0 represents the worst possible performance, and 100 represents the best possible performance. However, rescaling is highly sensitive to the choice of limits and extreme values (outliers) at both ends of the distribution. These outliers can unintentionally become thresholds, introducing artificial variability into the data. As a result, the selection of upper and lower bounds can impact the relative rankings of countries in the index.

To address this, a five-step decision tree was employed to determine the upper bound for each indicator:

(1) When specific quantitative thresholds are provided by the SDGs and their targets, those thresholds were used as the upper bound (e.g., zero poverty, universal school completion, universal access to water and sanitation, full gender equality).
(2) If no explicit SDG target is available, the principle of “leave no one behind” was applied, setting the upper bound to universal access or zero deprivation.
(3) For science-based targets that must be achieved by 2030 or later, these targets were used as the 100-point upper bound (e.g., zero CO₂ emissions by 2050 to meet the 1.5 °C climate goal, or 100 % sustainable fisheries management).
(4) If several countries have already exceeded an SDG target, the average of the top five performers was used as the upper bound (e.g., for child mortality rates).
(5) For all other indicators, the average of the top performers was used.

These principles treat the SDGs as “stretch targets” and focus attention on indicators where countries are lagging behind. The lower bound was defined at the 2.5th percentile of the distribution. Each indicator distribution was censored so that values exceeding the upper bound were scored 100, and values below the lower bound were scored 0.

### 2.4 Data description and sources

The Sustainable Development Goals (SDGs) are the 17 objectives established in the United Nations' 2030 Agenda for Sustainable Development, adopted in 2015, aimed at promoting global sustainable development [30]. The 17 goals encompass social, economic, and environmental aspects, including no poverty, zero hunger, good health and well-being, quality education, gender equality, clean water and sanitation, affordable and clean energy, decent work and economic growth, industry innovation and infrastructure, reduced inequalities, sustainable cities and communities, responsible consumption and production, climate action, life below water, life on land, peace, justice, and strong institutions, and partnerships for the goals [31]. Each SDG encompasses several specific sub-goals, totaling 169 in all. These goals cover various domains such as social, economic, and environmental aspects, aiming to achieve global sustainable development while maintaining human well-being, promoting economic prosperity, and protecting the environment. Each goal and sub-goal have corresponding indicators and monitoring methods for the assessment and monitoring of their achievement. Due to limited data access, this study selects 63 sub-objectives, as detailed in Appendix, which were the most obtainable indicators for measuring and assessing the sustainable development goals (SDGs) over the period of 2000–2019. These indicators were carefully chosen to cover the broad range of dimensions that align with the 17 SDGs. Additionally, while the study uses time-series data from 61 of these indicators, the data selection process was influenced by the availability and consistency of the indicators across the countries involved. We also acknowledge the potential impact of the COVID-19 pandemic on data accuracy, particularly in the years after 2020. Given the disruptions caused by the pandemic, some of the data from recent years may be subject to reporting inconsistencies, which could affect the precision of the analysis. For this reason, the study focuses on data up to 2019, ensuring that the analysis remains based on more reliable and consistent data.

The main data sources include: https://sdgs.un.org/, https://unstats.un.org/sdgs/, https://sdgdata.unep.org/, https://data.unicef.org/sdg/, https://open.undp.org/, https://www.who.int/data/gho/data/themes/sdg, http://www.fao.org/sustainable-development-goals/indicators/en/, https://unfccc.int/sdgs, http://emdat.be/country_profile/index.html, http://emdat.be/country_profile/index.html

### 2.5 Research framework diagram

**Fig. 1:**
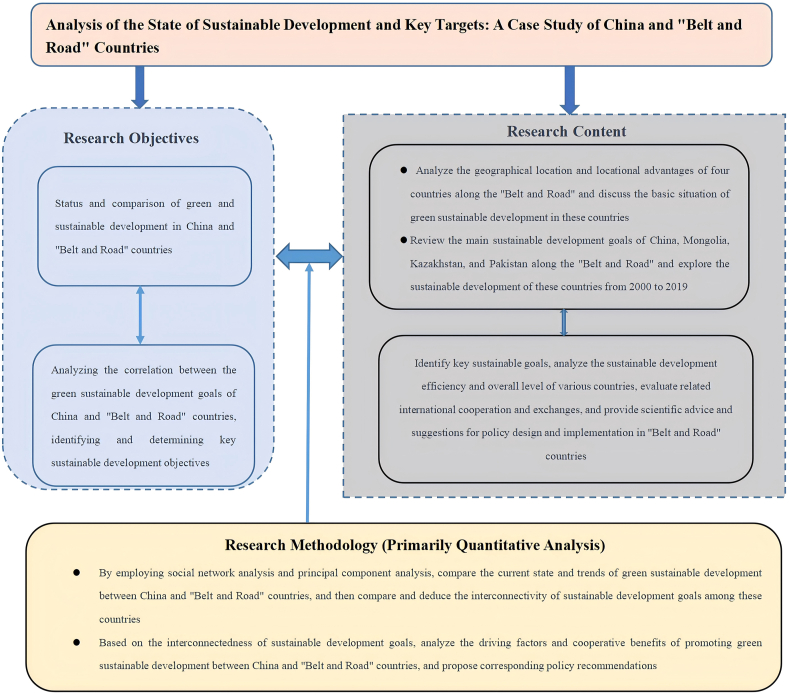
Research framework diagram.

Based on the above description of research methods, the research framework diagram for this study is as follows (Fig. 1):

## 3 Results

### 3.1 Introduction of “Belt and Road” countries

**Fig. 2:**
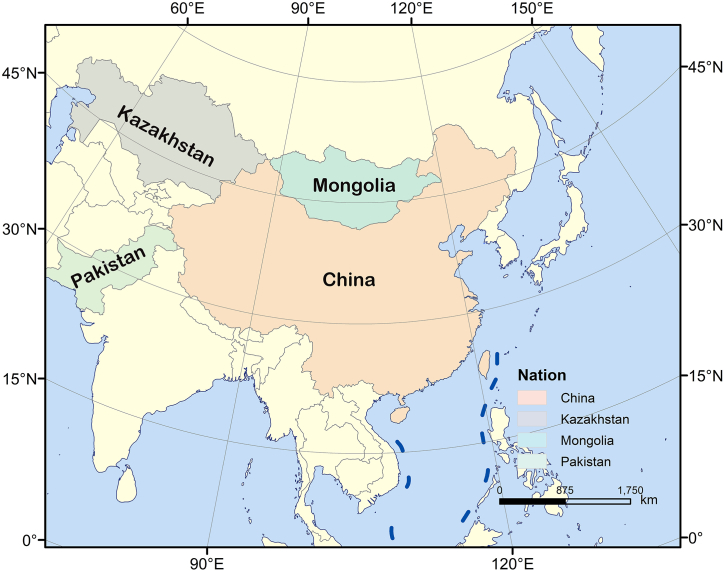
Geographic locations of China, Kazakhstan, Mongolia, and Pakistan.

As important partners in the “Belt and Road” initiative, China, Kazakhstan, Mongolia, and Pakistan are located in different regions along the “Belt and Road” route, with Fig. 2 illustrating the specific geographical locations of these four countries. China, as the initiator and a core country of the “Belt and Road”, connects the eastern and western ends of the Eurasian continent. Kazakhstan, a country in Central Asia, lying adjacent to China's western frontier, acts as one of the crucial routes from China to Europe, the Middle East, and South Asia. Mongolia, an important member of the China-Russia-Mongolia economic corridor, possessing access to the major markets of China and Russia, and also bordering western China, becomes a significant country in the northward extension of the “Belt and Road”. Pakistan, being an integral part of the China-Pakistan Economic Corridor -- a key project of the “Belt and Road” [32], stands as one of the vital junctions linking Central and South Asia, and one of the significant maritime entrances for China into the Indian Ocean.

Based on the principal component indicators and their economic significance, the study extracts the first and second principal components for “Belt and Road” Countries. From the principal component indicators, it is apparent that the first principal component represents " economic and social sustainable development ", which includes: (1) Health and Medical Services: Proportion of population using basic health services, prevalence of stunting in children under five, maternal mortality rate, under-five mortality rate by gender, death rate from cardiovascular diseases, cancer, diabetes, or chronic respiratory diseases, proportion of population using safely managed drinking water services, proportion of population with access to electricity, number of homicide victims per 100,000 people. (2) Social Equality and Political Participation: Proportion of women's seats in national parliaments, net amount of international development assistance, and received official aid. (3) Education: Completion rate of primary education, enrollment rate in preschool education, enrollment rate in higher education. (4) Economic Activities: Proportion of manufacturing value-added in GDP, share of labor force in GDP, total natural resource rents as a percentage of GDP, debt service as a percentage of exports of goods and services, annual GDP growth rate. The second principal component represents " environmental and social sustainable development ", which includes: (1) Resource Utilization and Environmental Protection: Proportion of forest area to land area, carbon dioxide emissions per unit of value-added, per capita material footprint, carbon dioxide emissions per unit of energy consumed, proportion of renewable energy in final energy consumption, recycling and recovery rate in waste management, proportion of local varieties at risk of extinction to those known to be at risk.(2) Welfare and Social Justice: Mortality rate from road traffic injuries, social attitudes towards women, proportion of women in employment, proportion of social assistance, number of sentenced prisoners, proportion of girls married or in union.

Utilizing formula (4), the total variance explained by the first and second principal components for each country is calculated to be 83.577 %, 70.815 %, 65.326 %, and 71.262 %, respectively, meeting the basic requirements for analyzing the sustainable development status of each country.

**Fig. 3:**
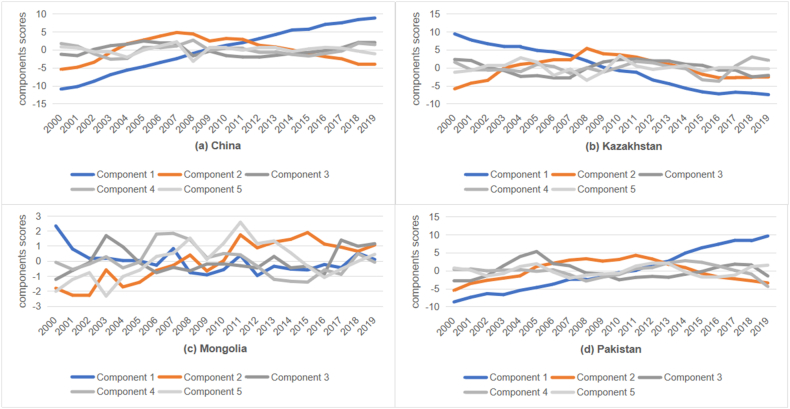
Comparison of sustainable development in (a) China, (b) Kazakhstan, (c) Mongolia, and (d) Pakistan from 2000 to 2019.

Fig.3(a–d) displays the situation of “economic and social sustainable development” (principal component 1) and “environmental and social sustainable development” (principal component 2) for “Belt and Road” Countries from 2000 to 2019.

In terms of “economic and social sustainable development”, China and Pakistan have seen a steady rise in scores, which may be associated with the relevant policies adopted by China and Pakistan. For instance, China has implemented comprehensive deepening reforms to make its market mechanisms more flexible and effective; Pakistan has implemented a series of economic reforms, such as reducing government intervention in the economy, promoting privatization, and improving the business environment. Mongolia saw a decline in “economic and social sustainable development” in the first ten years, followed by a gradual increase in the next decade. Kazakhstan's score has continued to decline, which may be related to its economic structure. Its economy is overly reliant on the oil and natural gas industries. This results in Kazakhstan's economy being highly dependent on the fluctuations of the international energy market [33]. Additionally, the country has faced issues such as political instability and systemic corruption over the past decades [34].

In terms of “environmental and social development”, aside from Mongolia's fluctuating score increase, China, Kazakhstan, and Pakistan have gone through a phase of development characterized by an initial increase and subsequent decrease. However, this does not imply that these countries have performed poorly in environmental sustainability in recent years. In fact, the “environmental and social development " indicators of these countries are often influenced by many other factors, such as sustainable social development, economic growth, population increase, and energy consumption. Similarly, the indicators of social sustainable development are also influenced by many other factors, such as cultural and political factors, etc. Nevertheless, the recent trend of declining scores in “environmental and social sustainable development” remains a cause for concern. Despite various policies and measures taken by these countries in environmental protection and social justice, the downward trend is still evident due to the impact of economic development, energy consumption, population growth, and other factors. Therefore, these countries need to enhance environmental awareness and resource utilization efficiency in the future, advance the development and use of renewable energy, and adopt reasonable measures to promote social equality and control population growth to achieve a more comprehensive sustainable development.

### 3.2 The achievement of sustainable development goals in four “Belt and Road” countries in 2022

**Fig. 4:**
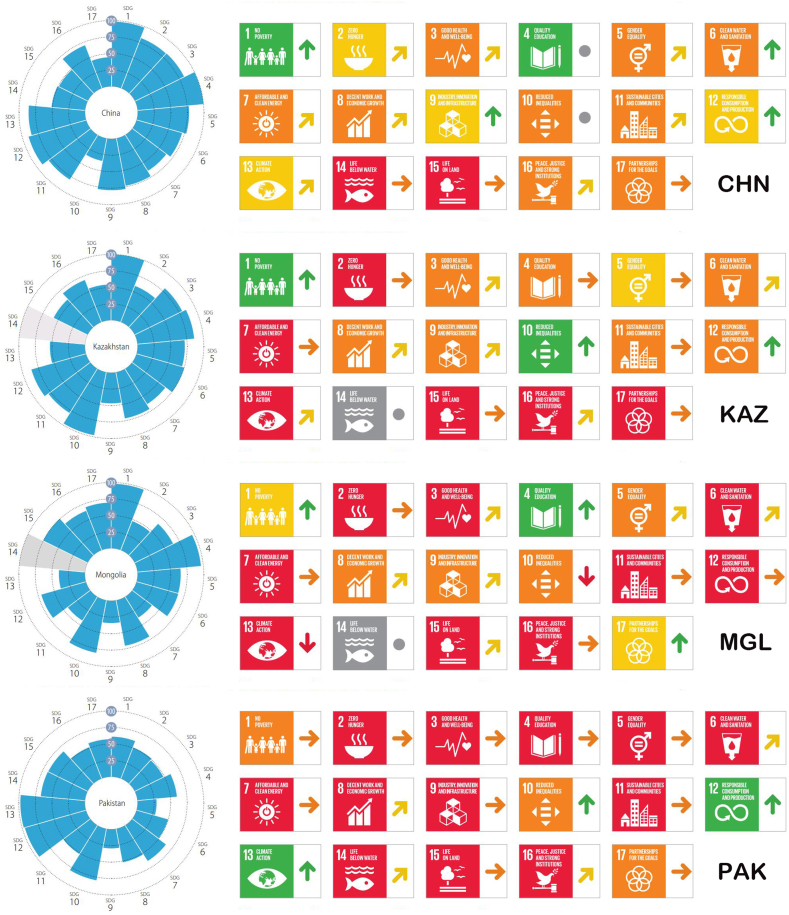
Ratings and Implementation Status of the 17 Sustainable Development Goals in “Belt and Road” Countries in 2022. *Levels:**Major Challenges**Significant Challenges**Challenges remain**SDG achieved**Information Unavailable. Trends:**Decreasing**Stagnating**Moderately improving**On track or maintaining SDG achievement**Information Unavailable. The abbreviations used in this figure represent the following countries: CHN: China; KAZ: Kazakhstan; MGL: Mongolia; PAK: Pakistan. Data Source:*https://dashboards.sdgindex.org/.

Based on the Sustainable Development Solutions Network (SDSN) and the Bertelsmann Foundation's computation of the individual and overall indices for Sustainable Development Goals [15], a comparison was made of the sustainable development status of “Belt and Road” Countries. Fig. 4 shows the rating and implementation of the 17 Sustainable Development Goals in 2022 for these countries.

From Fig. 4, it is evident that there are differences among countries in the implementation of sustainable development goals, particularly in terms of temporal and spatial differences in green sustainable development goals. Although these countries have common concerns and consensus on sustainable development, there is an urgent need to bolster international cooperation in the face of specific implementation challenges and development phases. By sharing successful practices, collaboratively researching solutions, and jointly advancing the growth and spread of green technologies, different countries can effectively tackle the challenges of sustainable development together. This form of cooperation not only serves to bridge disparities in time and space but also optimizes the strengths of each country, propelling the sustainable development agenda both regionally and worldwide.

### 3.3 Identification of key sustainable development goals for “Belt and Road” countries

**Fig. 5:**
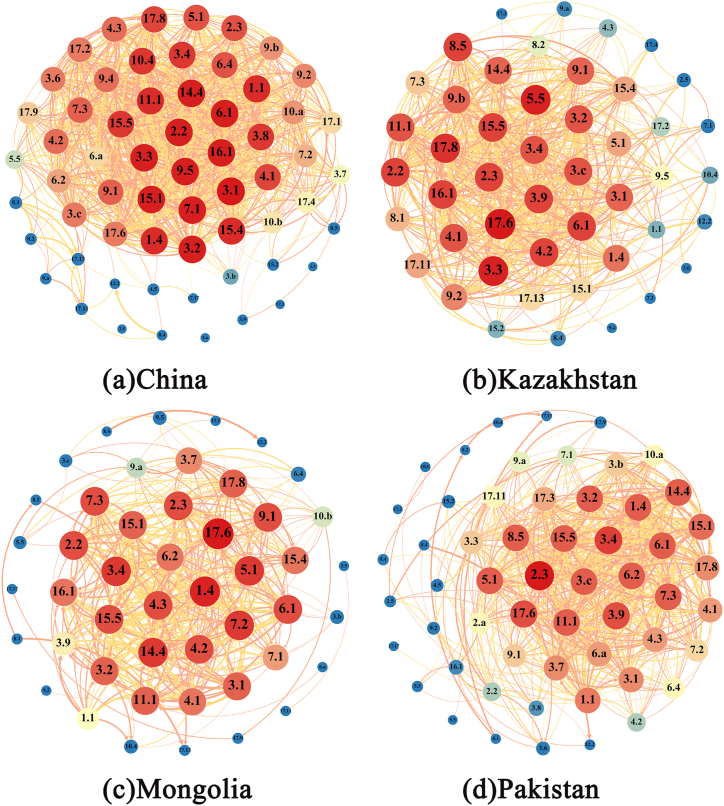
The correlation structure of 63 objectives in (a) China, (b) Kazakhstan, (c) Mongolia, and (d) Pakistan based on social network analysis.

Initially, raw data from four countries were collected, focusing on indicators for 169 sub-goals. Indicators with excessive missing data were excluded, and multiple imputation methods were used to supplement data for specific missing years. The gathered data was then standardized using the Z-score method, followed by a correlation analysis of the standardized data. Data calculations were performed in Gephi software to identify the subset of target data with the greatest impact on the entire network, as depicted in Fig. 5(a–d).

The color and size of the dots in the figure represent the importance level; the darker the color (such as red), the larger the circle, the higher the importance of the node; red and yellow colors indicate the direction of correlation, with red representing negative correlation and yellow positive correlation; the thickness of the edges represents the magnitude of correlation, the thicker the edge, the greater the correlation.

**Fig. 6:**
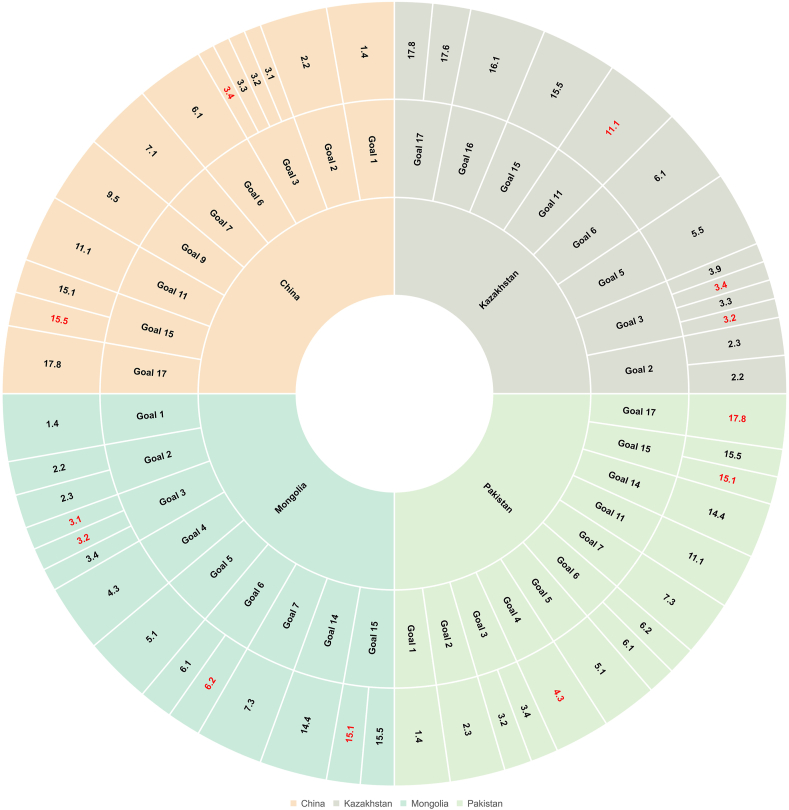
Key Sustainable Development Goals in “Belt and Road” **Countries.***Red indicates indices supplemented by principal component analysis, black indicates indices derived from social network analysis.*

In SPSS software, Principal Component Analysis (PCA) was conducted on the 63 Sustainable Development Goal (SDG) indicators, selecting the first few indicators with a significant variance contribution rate (greater than 5 %) as potential supplementary indicators. These supplementary indicators derived from the principal component analysis were added to the target data subset. The subset was then subjected to linear regression analysis against the extracted principal components, with the R^2^ value measuring the fit. The subset's influence on the principal components was thus interpreted. Finally, the most crucial sustainable development goal indicators were selected from these target variables. Fig. 6 displays the results for key sustainable development goals.

From Fig. 6, we can deduce that China's key sustainable development goals include promoting the construction of basic health facilities, enabling more people to access essential medical services(Goal 1–1.4), eradicating all forms of malnutrition(Goal 2–2.2), improving maternal health services, strengthening disease prevention and control, reducing maternal and neonatal mortality rates, enhancing medical infrastructure, improving sanitation facilities, promoting health education, reducing the incidence and mortality of infectious and non-communicable diseases(Goal 3–3.1, 3.2, 3.3, 3.4), ensuring universal and equitable access to safe and affordable drinking water(Goal 6–6.1), guaranteeing affordable and reliable modern energy services for all(Goal 7–7.1), encouraging innovation, enhancing scientific research, upgrading the technological capabilities of the industrial sector(Goal 9–9.5), developing inclusive, safe, resilient, and sustainable cities(Goal 11–11.1), protecting, restoring, and sustainably using terrestrial and inland freshwater ecosystems and their services, reducing the degradation of natural habitats, halting the loss of biodiversity(Goal 15–15.1, 15.5), promoting the application of information and technology, and strengthening the technology database(Goal 17–17.8).

The fundamental objectives of sustainable development in Kazakhstan focus on eliminating all types of malnutrition, enhancing the productivity of agriculture(Goal 2–2.2, 2.3), fostering the development of healthcare facilities, bettering hygiene infrastructure, spreading health education, lowering the mortality rate of newborns, diminishing the prevalence and fatalities due to infectious and non-infectious diseases, curtailing deaths and infections from chemicals(Goal 3–3.2, 3.3, 3.4, 3.9), ensuring comprehensive and effective involvement of women in decision-making processes at every level of political, economic, and public sectors(Goal 5–5.5), ensuring widespread and equitable access to safe and affordable drinking water(Goal 6–6.1), constructing cities that are inclusive, safe, capable of withstanding disasters, and sustainable(Goal 11–11.1), decreasing the degradation of natural habitats, halting the loss of biodiversity(Goal 15–15.5), reducing all forms of violence and associated mortality rates(Goal 16–16.1), intensifying international collaboration in the realms of science, technology, and innovation, endorsing the use of information and technology, and augmenting technological databases(Goal 17–17.6, 17.8).

Mongolia's key objectives in sustainable development include fostering the construction of basic sanitary infrastructure, making essential medical services more accessible to a larger population(Goal 1–1.4), eliminating all types of malnutrition, augmenting agricultural productivity(Goal 2–2.2, 2.3), enhancing healthcare services for pregnant and birthing women, bolstering disease prevention and control, lowering mortality rates of mothers and newborns, encouraging the development of healthcare facilities, bettering sanitation infrastructure, promoting health education, reducing the incidences and fatalities from non-communicable diseases(Goal 3–3.1, 3.2, 3.4), ensuring equal access to affordable and quality technical, vocational, and higher education for both genders(Goal 4–4.3), eradicating all forms of discrimination against women(Goal 5–5.1), ensuring widespread and equitable access to safe and affordable drinking water, providing proper and fair environmental and personal hygiene for all(Goal 6–6.1, 6.2), increasing the rate of energy efficiency improvement(Goal 7–7.3), sustainably managing fishery resources(Goal 14–14.4), protecting, restoring, and sustainably exploiting terrestrial and inland freshwater ecosystems and their services, diminishing the degradation of natural habitats, and preventing the loss of biodiversity(Goal 15–15.1, 15.5).

The principal objectives of sustainable development in Pakistan are to advance foundational healthcare infrastructure, granting increased access to essential medical services for more individuals(Goal 1–1.4), boosting agricultural productivity(Goal 2–2.3), fostering the development of healthcare facilities, enhancing sanitary conditions, disseminating health education, lowering rates of newborn mortality, reducing the incidence and fatalities of non-infectious diseases(Goal 3–3.2, 3.4), guaranteeing equal access for all to affordable, quality technical, vocational, and higher education(Goal 4–4.3), eradicating discrimination against women in all its forms(Goal 5–5.1), ensuring that everyone has universal and fair access to safe and affordable drinking water, providing suitable and equitable environmental and personal sanitation(Goal 6–6.1, 6.2), improving the rate of energy efficiency(Goal 7–7.3), constructing inclusive, secure, resilient, and sustainable urban areas(Goal 11–11.1), sustainably utilizing fisheries resources(Goal 14–14.4), protecting, rejuvenating, and sustainably exploiting terrestrial and inland freshwater ecosystems and their services, diminishing the degradation of natural habitats, halting the loss of biodiversity(Goal 15–15.1, 15.5), advancing the application of information and technology, and enhancing the technology database(Goal 17–17.8).

## 4 Discussion

According to the Sustainable Development Solutions Network (SDSN) and the Bertelsmann Foundation's calculation of the Sustainable Development Goals (SDGs) single index and overall index (SDSN, 2023), a scoring of the sustainable development status of “Belt and Road” Countries has been conducted to present a clearer and more direct view of each country's sustainable development status. The scoring results are shown in Table 1.

**Table 1:**
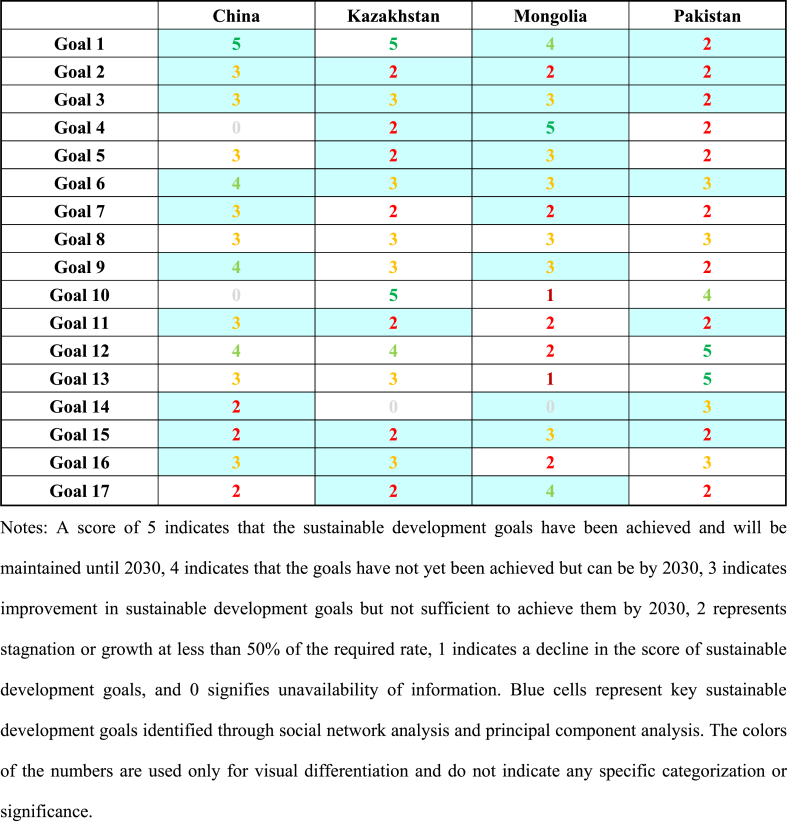
Scores of the current status of 17 sustainable development goals in “Belt and road” countries for 2022.

**Fig. 7:**
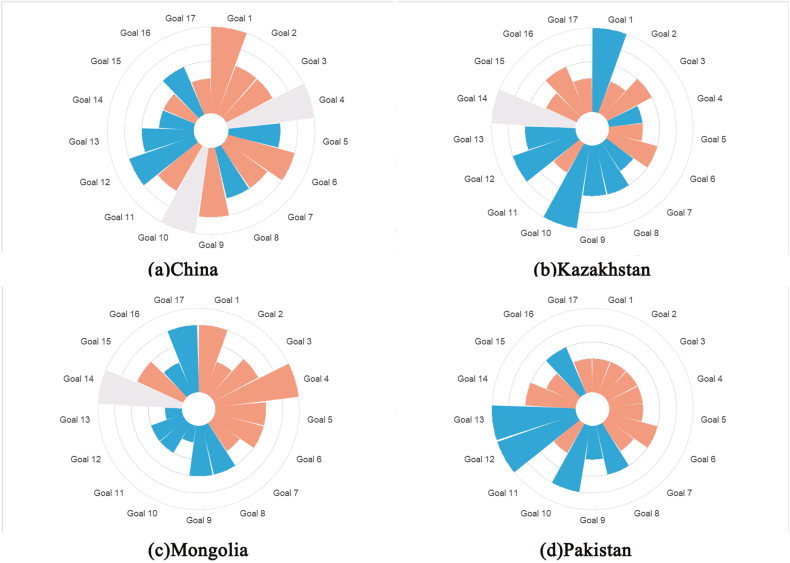
Sustainable Development Scores and Status of Key Sustainable Development Goals in (a) China, (b) Kazakhstan, (c) Mongolia, and (d) **Pakistan**. *Red represents key sustainable development goals; gray indicates missing data.*

To present the sustainable development scores and key sustainable development goals of the four countries more clearly and intuitively, we use a radar chart (Fig. 7(a–d)) for demonstration. It can be observed that China's key sustainable development goals, namely goals 1, 6, and 9, are expected to be achieved by 2030. Similarly, Mongolia is set to achieve goals 1 and 4 by 2030. However, Kazakhstan and Pakistan will not be able to achieve their key sustainable goals by 2030 and need to exert more effort and strengthen international cooperation and communication to promote the achievement of these goals. Overall, China shows good performance in achieving most of the sustainable development goals, but still needs improvement in ecological protection. Kazakhstan and Pakistan display similar overall conditions, showing weaknesses in technological innovation and affordable energy, and like China, they also have certain deficiencies in ecological protection. Mongolia shows weaker performance in aspects of sustainable development, such as in sustainable cities and communities, sustainable consumption and production, and in improving its response to climate change.

Taking Pakistan as an example, upon identifying key sustainable development goals, we find that Pakistan faces significant challenges in eradicating poverty (Goal 1). To achieve this goal, Pakistan can learn from other countries, especially from those like China and Kazakhstan that have already achieved Goal 1, adopting their experiences in rural development and infrastructure construction. Additionally, Pakistan can enhance trade and technology transfer with other countries, which will create more opportunities for Pakistan's economic and social development. Furthermore, in other key sustainable development areas such as education (Goal 4) and clean water and sanitation (Goal 6), Pakistan also needs to enhance international cooperation, learn from the experiences of other countries, and implement practical and effective measures. By engaging in international cooperation, Pakistan can more effectively realize its key sustainable development goals and foster the country's sustainable growth.

Additionally, through an in-depth analysis of Table 1, we can conclude that “Belt and Road” Countries share common key sustainable development goals in areas such as zero hunger (Goal 2), good health and well-being (Goal 3), clean water and sanitation (Goal 6), and life on land (Goal 15). This indicates that these four countries have similar development directions and shared aspirations in these areas, providing opportunities for cooperation in achieving these goals.

It is significant to observe that the four nations are mainly concentrated in areas related to green sustainable development concerning key sustainable development goals and their mutual links. Specifically, goals such as Goal 6 (clean water and sanitation), Goal 7 (affordable and clean energy), Goal 11 (sustainable cities and communities), and Goal 15 (life on land) pertain to areas like green energy, urban sustainable development, and ecosystem protection. This underscores the essentiality and importance of explicitly focusing on green sustainable development in the context of the “Belt and Road” initiative. For example, regarding clean water and sanitation: all four nations grapple with challenges of water shortage and pollution, requiring strengthened water resource management and water pollution treatment [35]. Particularly in rural and remote areas, the availability of clean water and sanitation facilities is comparatively low. These countries can strengthen cooperation in water resource management and pollution governance to collectively promote sustainable utilization of water resources and water environment protection [36]. Simultaneously, there can also be an increased investment in the construction of clean water and sanitation facilities in rural and remote areas. Regarding life on land: all four countries possess rich biodiversity resources but also face challenges of environmental degradation and biodiversity loss, needing strengthened ecosystem protection and biodiversity conservation [37]. These countries could enhance cooperation in ecosystem protection and biodiversity conservation, jointly promoting ecological environment protection and sustainable use.

From the above analysis, it is evident that fostering green sustainable development is in complete harmony with the critical sustainable development objectives of each country. In this context, strengthening international cooperation, sharing experiences and resources, particularly in the field of green sustainable development, will aid these countries in more effectively achieving their shared key sustainable development goals. Moreover, establishing a closer cooperation framework aligns with the long-term environmental development strategies of the “Belt and Road” countries, jointly promoting the realization of these key goals. Such cooperation not only helps promote sustainable development in each country but also contributes positively to the sustainable development of the entire region. In the process of achieving key sustainable goals in these countries, the construction of the “Belt and Road” provides important support and opportunities. For example, regarding Goal 7 (affordable and clean energy), the “Belt and Road” initiative can promote the dissemination and application of clean energy technologies, including solar, wind, and hydro power. These technologies help promote sustainable energy development in the countries along the route, thereby reducing carbon emissions and environmental pollution [38]. In addressing climate action (Goal 13), the initiative helps reduce reliance on traditional high-carbon energy sources, improve energy efficiency, and strengthen cooperation among countries along the route to jointly address climate change through promoting clean energy technology and reducing carbon emissions.

## 5 Conclusion

Through the analysis of the present situation, development trends, and correlations of the main sustainable development goals in the four countries, it becomes evident that cooperation for mutual development is an effective and key pathway to promote regional sustainable development. The “Belt and Road” initiative presents a viable platform for fostering sustainable development, enabling nations to thoroughly utilize this platform to intensify collaboration, exchange relevant experiences, and advance the realization of sustainable development objectives. Based on the analysis, the following conclusions can be drawn:

(1) In recent years, countries need to focus more on “environmental and social sustainable development”. Environmental and social issues not only affect people's survival and development but also directly impact the sustainability of the economy and future developmental prospects. To achieve sustainable development goals, countries along the “Belt and Road” should strengthen cooperation, promote resource and technology sharing, jointly advance the development and utilization of clean energy, control the emission of pollutants and waste management, and foster a sustainable process of urbanization and industrialization.
(2) China, Kazakhstan, Mongolia, and Pakistan each have their strengths and experiences in sustainable development, which can provide valuable lessons and references for other countries. By strengthening regional cooperation, these countries can jointly promote the process of sustainable development and contribute to global sustainable development. Investigating the interrelations of sustainable goals and identifying key sustainable objectives can further construct a cooperative system such as enhancing international scientific research collaboration: countries can establish joint laboratories, technology development centers, scientific infrastructure, etc., to collectively research issues in energy, environmental protection, climate change, and share results and technologies. Also, through scientific research collaboration, high-quality talent can be cultivated, promoting talent development and exchanges in the field of sustainable development; promoting transnational corporate cooperation: countries can build platforms for transnational corporate collaboration, through joint investments, and cooperative research and development, to advance industries related to sustainable development, such as new energy, clean technology, sustainable urban construction, and more. Simultaneously, multinational corporate cooperation also benefits the promotion of mutually beneficial economic collaboration between countries, fostering regional economic development; enhancing policy coordination: Countries can strengthen policy coordination in areas related to sustainable development, jointly research, formulate, and implement sustainable development strategies, policies, and standards to advance the sustainable development process. Policy coordination can also promote political and economic collaboration in the region, enhancing mutual trust and friendship; encouraging participation of social organizations: countries can motivate social organizations to actively participate in sustainable development practices and promotion, such as NGOs and foundations. Through the participation of social organizations, collaboration among governments, businesses, and the public can be facilitated, advancing various aspects of sustainable development such as environmental protection, social justice, and poverty eradication.
(3) The “Belt and Road” initiative has greatly aided countries in achieving their sustainable development goals. The initiator of this initiative, China, has set forth the grand objective of promoting shared development and is committed to achieving win-win development through enhanced cooperation with countries along the route. By identifying their key sustainable development goals, countries can better understand their development needs and consider the allocation of resources more judiciously, thereby achieving more efficient international cooperation. Furthermore, key sustainable development goals provide an opportunity for international cooperation among countries, where different nations can share experiences and technologies to jointly advance these goals, further promoting global sustainable development. The construction of the “Belt and Road” initiative provides more opportunities and possibilities for cooperation among countries, advancing the achievement of sustainable development goals, especially in green development, and making a significant contribution to global sustainable development.

## 6 Limitations and future research directions

As noted in the methodology, the limited availability of reliable and consistent data for certain indicators has been a key challenge in this study. For example, some SDGs lacked sufficient data across all four countries, leading to the selection of 63 obtainable sub-indicators. These indicators, while representative, may not fully capture the breadth of each SDG target. Additionally, data gaps and inconsistencies, particularly in the years following 2020, presented further challenges. The COVID-19 pandemic disrupted data collection and reporting, leading to potential inaccuracies in post-2019 data. Given these disruptions, we focused on data up to 2019 to ensure the accuracy and reliability of the analysis.

Moving forward, we envision that as data availability improves, a more extensive and robust analysis can be conducted. Future research should incorporate post-2019 data to provide a more up-to-date assessment of sustainable development trends. This would help account for the disruptions caused by the COVID-19 pandemic and improve the overall data quality. Additionally, we recommend addressing potential challenges such as indicator variability, missing data, and imputation techniques to refine future analyses.

Further, classifying countries based on their development stages and regional characteristics could offer more nuanced insights. This approach would help identify targeted areas for collaboration, especially in the context of the Belt and Road Initiative. A more detailed classification could also reveal how countries' SDG priorities align and where opportunities for mutual benefit and cooperation might exist. By exploring these avenues, future research can provide valuable insights and foster stronger regional and international collaboration.

## Funding information

This work was funded by the 10.13039/501100018625Shanghai Science and Technology Commission (23ZR1404100), the Fudan-USyd Ignition Grants (grant no. 7307557635), the Sino-673 German Center (M-0049), and Fudan Tyndall Centre of Fudan University (IDH6286315).

## CRediT authorship contribution statement

**Shun Chen:** Writing – original draft, Visualization, Project administration, Methodology, Investigation, Data curation, Conceptualization. **Ping Jiang:** Writing – review & editing, Supervision, Investigation, Conceptualization. **Pin Zuo:** Writing – review & editing, Validation. **Zolboo Dashnyam:** Writing – review & editing, Validation.

## Informed consent

This article does not contain any studies with human participants performed by any of the authors.

## Data availability

The data that support the findings of this study are available on request from the corresponding author, upon reasonable request.

## Ethical approval

This article does not contain any studies with human participants performed by any of the authors.

## Declaration of competing interest

The authors declare that they have no known competing financial interests or personal relationships that could have appeared to influence the work reported in this paper.
