# Supplementary material for: Analysis of the state of sustainable development and key targets: A case study of China and “Belt and Road” countries
Source: Heliyon. 2025 Jan 28;11(3):e42305. doi: 10.1016/j.heliyon.2025.e42305 (PMC11850173; doi:10.1016/j.heliyon.2025.e42305)
Supplement: Multimedia component 1 [file mmc1.docx]

**Table 1**

63 Sub-Objectives Selected under the Sustainable Development Goals Framework.

| ID | Indicator Name |
| --- | --- |
| 1.1 | Poverty headcount ratio at $1.90 a day (2011 PPP) (% of population) |
| 1.4 | Proportion of population using basic sanitation services (%) |
| 1.5、11.5、13.1 | Number of deaths due to disaster (number) |
| 2.2 | Proportion of children moderately or severely stunted (%) |
| 2.3 | Agriculture value added per worker (constant 2010 US$) |
| 2.5 | Proportion of local breeds classified as being at risk as a share of local breeds with known level of extinction risk (%) |
| 2.a | Total official flows (disbursements) for agriculture, by recipient countries (millions of constant 2020 United States dollars) |
| 3.1 | Maternal mortality ratio (modeled estimate, per 100,000 live births) |
| 3.2 | Under-five mortality rate, by sex (deaths per 1,000 live births) |
| 3.3 | Tuberculosis incidence (per 100,000 population) |
| 3.4 | Mortality rate attributed to cardiovascular disease, cancer, diabetes or chronic respiratory disease (probability) |
| 3.6 | Death rate due to road traffic injuries, by sex (per 100,000 population) |
| 3.7 | Adolescent fertility rate (births per 1,000 women ages 15-19) |
| 3.8 | Immunization, DPT (% of children ages 12-23 months) |
| 3.9 | Mortality rate attributed to unintentional poisonings, by sex (deaths per 100,000 population) |
| 3.b | Total official development assistance to medical research and basic heath sectors, gross disbursement, by recipient countries (millions of constant 2020 United States dollars) |
| 3.c | Physicians (per 1,000 people) |
| 4.1 | Primary completion rate, total (% of relevant age group) |
| 4.2 | School enrollment, preprimary (% gross) |
| 4.3 | School enrollment, tertiary (% gross) |
| 4.5 | School enrollment, secondary (gross), gender parity index (GPI) |
| 5.1 | Women Business and the Law Index Score (scale 1-100) |
| 5.5 | Proportion of seats held by women in national parliaments (%) |
| 6.1 | People using at least basic drinking water services (% of population) |
| 6.2 | Proportion of population practicing open defecation (%) |
| 6.4 | Level of water stress: freshwater withdrawal as a proportion of available freshwater resources (%) |
| 6.a | Total official development assistance (gross disbursement) for water supply and sanitation, by recipient countries (millions of constant 2020 United States dollars) |
| 7.1 | Proportion of population with access to electricity (%) |
| 7.2 | Renewable energy share in the total final energy consumption (%) |
| 7.3 | Energy intensity level of primary energy (megajoules per constant 2017 purchasing power parity GDP) |
| 8.1 | Annual growth rate of real GDP per capita (%) |
| 8.2 | Annual growth rate of real GDP per employed person (%) |
| 8.4 | Material footprint per capita |
| 8.5 | Unemployment, total (% of total labor force) (modeled ILO estimate) |
| 9.1 | Air transport, passengers carried |
| 9.2 | Manufacturing value added (constant 2015 United States dollars) as a proportion of GDP (%) |
| 9.4 | Carbon dioxide emissions per unit of manufacturing value added (kilogrammes of CO2 per constant 2015 United States dollars) |
| 9.5 | Research and development expenditure as a proportion of GDP (%) |
| 9.a | Total official flows for infrastructure, by recipient countries (millions of constant 2020 United States dollars) |
| 9.b | Proportion of medium and high-tech manufacturing value added in total value added (%) |
| 10.4 | Labour share of GDP (%) |
| 10.a | Tariff rate, applied, simple mean, all products (%) |
| 10.b | Total assistance for development, by recipient countries (millions of current United States dollars) |
| 11.1 | Urban population (% of total population) |
| 12.2 | Total natural resources rents (% of GDP) |
| 14.4 | Total fisheries production (metric tons) |
| 15.1 | Forest area (% of land area) |
| 15.2 | Tree cover loss |
| 15.4 | Average proportion of Mountain Key Biodiversity Areas (KBAs) covered by protected areas (%) |
| 15.5 | Red List Index |
| 16.1 | Intentional homicides (per 100,000 people) |
| 17.1 | Total government revenue (budgetary central government) as a proportion of GDP (%) |
| 17.2 | Net official development assistance and official aid received (current US$) |
| 17.3 | Personal remittances, received (% of GDP) |
| 17.4 | Debt service as a proportion of exports of goods and services (%) |
| 17.6 | Fixed broadband subscriptions per 100 inhabitants, by speed (per 100 inhabitants) |
| 17.8 | Proportion of individuals using the Internet (%) |
| 17.9 | Total official development assistance (gross disbursement) for technical cooperation (millions of 2020 United States dollars) |
| 17.11 | Exports of goods and services (% of GDP) |
| 17.13 | Annual GDP growth (%) |
| 17.17 | Amount of United States dollars committed to public-private partnerships for infrastructure, million USD real |

Data Source: <https://sdgs.un.org/>, https://databank.worldbank.org/reports.aspx?source=sustainable-development-goals-(sdgs).
